# Detection of Self-Harm in Electronic Mental Health Records Using Privacy-Preserving Local Language Models: Methodological Study

**DOI:** 10.2196/87586

**Published:** 2026-06-02

**Authors:** Andrey Kormilitzin, Dan W Joyce, Apostolos Tsiachristas, Rohan Borschmann, Navneet Kapur, Galit Geulayov

**Affiliations:** 1 Department of Psychiatry, University of Oxford, Warneford Hospital, Warneford Lane, Oxford, England, OX3 7JX, United Kingdom, 44 01865305337; 2 NIHR Oxford Health Biomedical Research Centre, Oxford, United Kingdom; 3 Institute of Population Health, University of Liverpool, Liverpool, England, United Kingdom; 4 Mental Health Research for Innovation Centre, University of Liverpool, Liverpool, England, United Kingdom; 5 Mersey Care NHS Foundation Trust, Liverpool, England, United Kingdom; 6 Nuffield Department of Primary Care Health Sciences, University of Oxford, Oxford, England, United Kingdom; 7 Health Service and Population Research Department, Institute of Psychiatry, Psychology & Neuroscience, King’s College London, London, United Kingdom; 8 Justice Health Group, School of Population Health, Curtin University, Perth, Western Australia, Australia; 9 Melbourne School of Psychological Sciences, The University of Melbourne, Melbourne, Victoria, Australia; 10 Division of Psychology and Mental Health and NIHR Greater Manchester Patient Safety Research Collaboration, University of Manchester, Manchester, England, United Kingdom

**Keywords:** self-harm, electronic health records, large language models, privacy, Gemma3, Ollama, temporal information extraction

## Abstract

**Background:**

Self-harm is the strongest risk factor for suicide and an important outcome for mental health care. Although prevalent in clinical populations, it is often imprecisely captured in routinely collected clinical data, where it is often recorded and stored as unstructured free text. Contemporary language models, such as GPT (OpenAI) and Gemini (Google), can analyze free-text clinical notes, but such models may violate data governance of processing sensitive patient data.

**Objective:**

This study aimed to evaluate whether a privacy-preserving language model running entirely within an institution’s secure computing infrastructure (here, the UK National Health Service [NHS]) could accurately identify the presence and timing of self-harm using electronic health records from secondary mental health care.

**Methods:**

Clinical notes were drawn from Oxford Health NHS Foundation Trust using a multistage workflow: (1) a random sample of 1000 patients with a psychiatric diagnosis, defined according to the *ICD-10* (*International Statistical Classification of Diseases, Tenth Revision*; codes F00–F99); (2) candidate-note identification using a Gemma3-4b language model to flag notes containing self-harm content; and (3) from those candidates, 1352 randomly sampled notes were selected for expert annotation, resulting in gold-standard corpus enriched for self-harm content. Clinical notes were annotated for the presence of self-harm and its timing (≤90 days, >90 days, or unknown). A privacy-preserving locally served 27-billion-parameter Gemma 3 language model (“Gemma3-27b”) was used as the core model. Prompts were systematically developed and refined using a labeled development set to identify self-harm and generate a structured output per clinical record. Gemma3-27b performance was compared against a strong baseline multilabel text classification model based on robustly optimized BERT pretraining approach (RoBERTa), a transformer-based language model architecture. Model performance was evaluated using precision, recall, and the *F*_1_-score (harmonic mean of precision and recall), with 95% CIs estimated from 1000 bootstrap samples with replacement.

**Results:**

Gemma3-27b outperformed the RoBERTa classifier across all categories, achieving Precision=0.92, Recall=0.92 (sensitivity), and *F*_1_-score=0.92 for notes containing self-harm, and Precision=0.97, Recall=0.97 (specificity), and *F*_1_-score=0.97 for notes without self-harm. For the 51 notes labeled as recent self-harm in the held-out test set, Gemma3-27b achieved Precision=0.84, Recall=0.75, and *F*_1_-score=0.79. The global weighted *F*_1_-score of Gemma3-27b across all categories was 0.88, compared to 0.85 for RoBERTa.

**Conclusions:**

With systematic prompt development on a labeled development set, but no gradient-based fine-tuning, the current Gemma3-27b language model matched or exceeded a fine-tuned RoBERTa classifier for ascertaining self-harm events and their timing. Aggregate gains were modest, while improvements were largest in the most challenging, lower-frequency timing categories. On a simplified binary recent-versus-other task, RoBERTa performed marginally better, indicating that supervised classifiers remain highly effective when the task is simplified and sufficient labeled data exist. This work demonstrates the technical feasibility of privacy-preserving self-harm detection within a secure NHS research environment.

## Introduction

### Background

Self-harm (intentional self-poisoning or self-injury, irrespective of motivation [1]) represents a major public health challenge. In England, approximately 5000 individuals die by suicide each year [2] and more than 200,000 individuals present to general hospitals due to self-harm [3]. Many more self-harm without seeking treatment [4].

Self-harm is the strongest risk factor for suicide [5]. Individuals who present to clinical services following self-harm are over 100 times more likely to die by suicide compared to those who do not self-harm [6,7]. Their risk of accidental death and death by natural causes is also markedly elevated [5]. Furthermore, these individuals have a higher risk of further nonfatal self-harm and adverse psychosocial outcomes [6,8,9].

Despite it being an important outcome for mental health care, information about self-harm is often imprecisely captured in many health care settings. For example, in one study from England [10], the investigators compared research-derived rates of hospital-presenting self-harm to official hospital episode statistics [11] data. The study found substantial under-ascertainment in official statistics compared with the research-derived figures, even though both sources drew on the same underlying clinical information. Accurate and systematic identification of self-harm across settings is essential for conducting valid and reliable research and for planning and delivering effective intervention strategies.

Suicide and self-harm research involves numerous methodological challenges. It can be resource-intensive and costly, with data collected and collated over many years from some (but not all) relevant settings. Consequently, many instances of self-harm go undetected, leading to missed opportunities for intervention and compromised research. Leveraging existing data collected as part of routine patient care can provide a valuable, contemporaneous, and economical source of information. Such data, which contain a wealth of information, have been used to study many health conditions, for example, cardiovascular disease, diabetes, and osteoarthritis [12,13]. However, using such rich information comes with significant challenges, particularly due to the large volume of data, much of which is often collected and stored in an unstructured narrative format. Advances in artificial intelligence and natural language processing (NLP) present an opportunity to unlock, retrieve, and convert this information into a format accessible for research and clinical care.

Previously, investigators have used the Clinical Record Interactive Search (CRIS) database of the South London and Maudsley National Health Service (NHS) Foundation Trust to identify suicidal ideation and self-harm from free text in secondary mental health electronic health records (EHRs) [14]. Such models show good performance in identifying patients who have self-harmed. Identifying the timing of the self-harm through free text, however, has been more challenging [15]. The timing of self-harm is important for both research and clinical practice. Evaluating the effect of interventions or routine care depends on accurately establishing the timing of self-harm. Similarly, reliable longitudinal analysis relies on ascertaining the temporal sequence of self-harm alongside its covariates. Importantly, the risk of suicide and repeat self-harm is acutely elevated soon after a self-harm episode [7,16]. As such, accurately capturing the timing of self-harm episodes is critical for identifying individuals in need of timely interventions and risk reduction strategies.

### Machine Learning for Self-Harm Identification

Well-established machine learning models for typical NLP tasks, such as named-entity recognition, relationship extraction, text classification tasks, and negation detection, have shown good ability to identify and structure the concepts of interest [17]. However, training such models relies on a large amount of data, manually annotated by experts. Collecting a sufficient amount of high-quality annotated data can be an expensive and time-consuming task. Since the introduction of large language models (LLMs) and, in particular, GPT and their chatbot interface, such as ChatGPT, the information extraction field has seen a paradigm shift. Multiple studies have shown that generic LLMs (eg, GPT, Claude, and Gemini) trained on a massive corpus from the internet can identify concepts of interest and generate a structured output following the prompt tailored for each particular task [18]. For instance, LLMs have been successfully applied to extract complex relationships between biomedical entities from the scientific literature by carefully prompting the model with a description of the desired relationship [19]. Furthermore, LLMs have demonstrated strong performance in extracting events and their context from news articles, outperforming traditional supervised models in some cases, especially in low-resource settings [20]. Additionally, recent work has explored the use of LLMs for extracting information from noisy and ambiguous user-generated content like social media posts, showing promising results in identifying relevant entities and topics despite the informal language and varied writing styles [21].

However, the use of proprietary LLM services via their application programming interface (API), such as those provided by OpenAI (GPT), Anthropic (Claude), and Google (Gemini), poses significant challenges to patient data privacy and may not be compliant with clinical information governance. In contrast, if an LLM can be implemented within a health care institution’s own secure clinical data environment, there is no need to risk exposing sensitive and confidential data via APIs to proprietary services. Until recently, implementing LLMs (including training and inference) has been implausible because of their memory and computing costs. With the introduction of quantized LLMs, models that have been made smaller and more computationally efficient by storing the numerical values of their parameters in a simpler form, open-weight models such as Gemma3-27b can be hosted and used for inference on modest compute resources with performance (for specific tasks) only marginally lower than the original (not quantized) model. Therefore, researchers have explored the use of these local versions of LLMs, such as Llama [22] for information extraction from clinical records [23,24], as well as for identifying acts of suicidality [25].

### Motivation and Our Contribution

In this study, we evaluated whether privacy-preserving local language models can identify self-harm and its timing in secondary mental health records, converting unstructured clinical notes into structured data. We assessed the semantic reasoning capabilities of a pretrained language model to distinguish self-harm events from related concepts (eg, ideation and risk assessments) and classify their timing. Specifically, we tested whether an open-weight Gemma3 model with 27 billion parameters, deployed locally, can accurately detect self-harm and identify its timing without gradient-based fine-tuning (ie, without updating the model’s internal parameters on our data), relying instead on systematic prompt development using a labeled development set.

We compared the current approach against a supervised robustly optimized BERT pretraining approach (RoBERTa) classifier (a commonly used model) trained on identical data. Model performance was assessed using precision, recall, and the *F*_1_-score (the harmonic mean of precision and recall) with 95% CIs estimated using 1000 bootstrap samples with replacement. We hypothesized that local language models would (1) match or exceed supervised model performance through prompt-based inference guided by a labeled development set, thereby reducing the volume of annotated data needed for gradient-based training; and (2) mitigate data governance barriers inherent in using cloud-based solutions via APIs, enabling deployment within health care institutions.

This work addresses the critical need for accurate self-harm identification in clinical records, using language models that can be deployed locally under strict patient confidentiality standards for sensitive mental health data and within constrained computational resources.

## Methods

### Definition of Self-Harm

Self-harm refers to any form of intentional self-poisoning or self-injury, irrespective of motivation [1]. It can take many forms, including overdosing on medications, ingesting a non-ingestible substance, or inflicting injury upon oneself through actions like cutting. In clinical settings (eg, hospital emergency departments and mental health services), self-harm ascertainment relies on a clinician’s judgment; ie, a clinician will determine whether the self-inflicted act was intentional, as opposed to accidental, even in the absence of patient confirmation [26].

### Data Source and Ethics

Data for this study were sourced from the CRIS system by the Akrivia Health [27] analytics platform on behalf of the Oxford Health NHS Foundation Trust, UK. Akrivia Health [27] provides a secure research environment with a robust information governance framework compliant with national statutory regulations for health care data. The CRIS database comprises pseudonymized EHRs including free-text clinical notes as well as structured data fields from secondary care mental health services [28]. Studies using this platform require approval from the health care institution that provided the data.

### Cohort Selection

**Figure 1.**
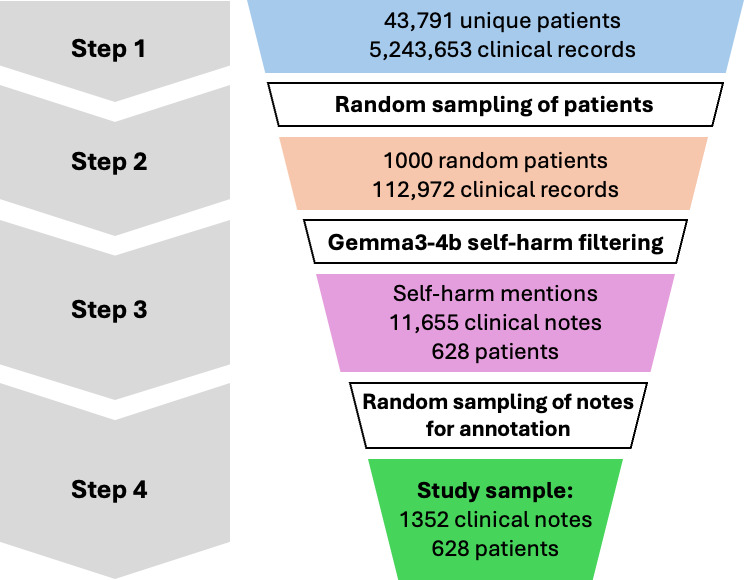
The data flow diagram of the clinical notes used to develop the models.

The study population involved individuals aged 18 years or older with a confirmed psychiatric diagnosis (see Section A of Multimedia Appendix 1) according to the *ICD-10* (*International Statistical Classification of Diseases, Tenth Revision*), who were in contact with specialist secondary mental health care services. Clinical records of patients with primary diagnoses of *ICD-10* codes F00-F99: Mental and Behavioral Disorders (see Section A of Multimedia Appendix 1), recorded between March 1, 2016, and March 1, 2022 (inclusive), were randomly sampled for annotation, as described in Figure 1.

In contrast to a common approach of identifying clinical notes that contain mentions of self-harm using a key-word fuzzy pattern matching (which can be biased in identifying cases beyond the predefined keywords), we opted for using a small, yet capable LM (“Gemma3”) with 4 billion parameters (“Gemma3-4b”) prompted to identify potential self-harm events minimizing the risk of the aforementioned key word-based bias (see Section B of Multimedia Appendix 1).

Figure 1 provides an overview of the end-to-end data selection process in this study. First, an initial cohort of 43,791 patients with over 5.2 million clinical notes satisfying the above criteria was extracted from the Oxford Health NHS CRIS database (Step 1). Given the limited human annotator resources, we randomly sampled 1000 patients (Step 2), followed by retrieving clinical notes containing potential mentions of self-harm using a lightweight “Gemma3-4b” LM (Step 3). However, the model identified over 11,000 notes containing self-harm, which significantly exceeded the capacity of our clinical annotators. As such, we further randomly reduced the cohort of 11,655 clinical notes containing self-harm to 1352 clinical notes from 628 unique patients (Step 4).

Since we used the Gemma3-4b language model to initially identify clinical notes that might contain self-harm cases, the text we found was richer in this type of content than one would find across all patient records. To mitigate this selection bias, we further randomly sampled 1352 notes from 628 different patients for expert review. While our annotated dataset likely contains more self-harm language than a typical sample would, the random selection process made sure we weren’t inadvertently favoring particular patients, specific time frames, or certain diagnostic groups.

This enrichment improves annotation efficiency by increasing the proportion of positive examples available for model development, but it changes the class distribution relative to routine care. In particular, model performance metrics, for example, positive predictive value may differ when applied to all clinical notes, where the prevalence of self-harm mentions is substantially lower. Implications for routine deployment are discussed in the Limitations section.

### An Annotation Schema and a Curated Dataset

Manual annotation of textual data is essential for developing and evaluating NLP models for information extraction. A systematic annotation process involving expert coders ensures unambiguous tagging of text segments according to a predefined schema, enabling models to learn meaningful patterns and providing a human benchmark for performance.

We used a multilabel annotation schema focusing on whether an actual act of self-harm occurred and whether it was (1) recent (occurring within 90 days of documentation), (2) historical (those occurring >90 days prior to documentation), or (3) of unknown timing (the timing could not be determined). Specifically, if annotators identified a self-harm event, the corresponding text was labeled as “Self-harm present” along with a timing tag. Where it was not possible to unambiguously determine whether a self-harm event took place, we labeled these cases as “unknown self-harm.” Clinical notes that did not include self-harm events (eg, a clinical note describing a patient with psychotic symptoms with no mentions of self-harm or one that mentions risk of self-harm but not actual self-harm) were unlabeled. The annotation protocol included initial training and calibration sessions, detailed guidelines with clinical examples, and regular interrater reliability assessments. Disagreements were jointly reviewed to refine decision boundaries and for consistency. The schema thus comprises 5 distinct labels as shown in Table 1.

**Table 1.** Five categories to designate the self-harm events used in the study.

Label	Explanation
Self-harm absent	Statement negating self-harm or where self-harm was not mentioned at all (eg, a clinical note recording only psychotic symptoms).
Self-harm present	Explicit description of a self-harm (eg, self-poisoning or self-injury) event by the patient.
Recent	Event occurred within the last 90 days prior to the note date.
Historical	Event occurred more than 90 days prior to the note date.
Unknown timing	Where time cannot be determined from the information provided (eg, “the patient has self-harmed previously” or “Deliberate self-harm scars and burns evident”).

All forms of intentional self-inflicted harm (including suicide attempts and self-harm where the specific motivation was not explicitly mentioned) were in scope; self-harm ideation (eg, “patient feels like cutting,” and “patient wishes to end it all”) were excluded unless a self-harm act was also mentioned.

Many patients had a long-documented history of contact with secondary mental health services. Annotators were instructed to treat each clinical note extract as a standalone document, independent of any decisions made about previous extracts from the same patient.

### Partitioning of Data for Training and Validation

**Figure 2.**
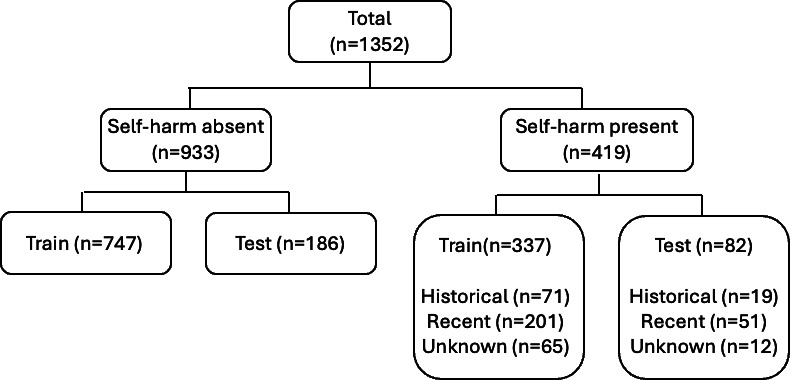
The distribution of 1352 clinical notes in the gold standard corpus according to self-harm status and timing labels, by training (80%) and testing (20%) sets.

The annotated sample of 1352 examples was split at the patient level, to avoid data leakage, into the training (80%, n=1084) and test (20%, n=268) sets, respectively. Resulting class distribution within the training and test splits is summarized in Figure 2. In most instances, when self-harm was mentioned by a clinician, there was sufficient information to determine whether this was a recent or past event.

### Annotation Procedure

In order to evaluate the developed annotation schema and the degree of agreement, 2 data annotators, including 1 researcher (LB) with over 30 years’ experience collecting and coding self-harm information from hospital records in the United Kingdom, and another (GG) with more than 15 years’ experience researching self-harm and suicidal behavior, refined category boundaries and created a decision flowchart. They subsequently independently further labeled 1352 randomly sampled notes.

### Comparing the Agreement on Annotations

Interannotator agreement was calculated on a sample of 160 notes annotated by two raters independently, using a Cohen κ hierarchical approach. Each clinical note was annotated along two categories: (1) self-harm status (“present” or “absent”) and (2) timing of self-harm (“recent,” “historical,” or “unknown”). The timing labels were sought only if self-harm was “present.” As the second decision category (ie, timing of self-harm) was conditional on the first category, we opted to report interannotator agreement using a hierarchical protocol: (1) assess agreement on self-harm status (present or absent); (2) assess agreement on the timing of self-harm event (“recent,” “historical,” or “unknown”) within the subset of notes where both annotators agreed that self-harm was present.

For category one—self-harm present or absent—each note contributed to a 2-by-2 contingency table and the resulting *κ_self-harm_* captured chance-corrected concordance on event detection. Using the subset of notes where both coders marked self-harm present, each annotator assigned 1 of 3 nominal categories: recent, historical, or unknown, yielding a 3-by-3 table from which an unweighted *κ_recency_* was computed. The 3-by-3 structure reflects the 3 possible timing categories assigned independently by each of the 2 annotators. Uncertainty estimates for both *κ* coefficients were obtained via 1000-fold stratified bootstrap resampling with replacement from the 160 jointly annotated notes. At each iteration, the set of notes was resampled while preserving the marginal class distribution (ie, the proportion of “present” vs “absent” for self-harm status, and the relative frequencies of “recent,” “historical,” and “unknown” within the subset marked as self-harm present). For each bootstrap replicate, *κ* was recalculated, and the 2.5th and 97.5th percentiles of the resulting empirical distribution were used to form 95% CIs. This stratification ensured that class imbalance did not distort the variability estimates of *κ*.

### Model Development

### Core Language Model

While language models have demonstrated strong capabilities in clinical text processing and reasoning (Wei et al [29] and Huang et al [30]), their computational demands can be prohibitive [31]. Therefore, we explored the Gemma3-27b, a decoder-only transformer model with 27 billion parameters and a 128K token context window [32]. The model is based on a novel architecture with a 5:1 ratio of local to global attention layers, where local layers use sliding window attention over 1024 tokens to reduce memory consumption during inference. The model was quantized to 4-bit precision (Q4_K_M format), resulting in a 10.6 GB model size and served locally within a secure environment at the Oxford Health NHS Foundation Trust using the Ollama framework (v0.9.6) with llama.cpp backend [33]. All experiments were conducted on the Microsoft Azure T4 instance (“Standard_NC8as_T4_v3”) with 16GB graphical processing unit (GPU) memory as a cost-effective solution for the NHS for information extraction and reasoning tasks.

### Baseline Multilabel Text Classification

To evaluate the benefit of language models for semantic reasoning on self-harm, we trained and evaluated a transformers-based text classification model as a baseline (a benchmark model). We chose the RoBERTa model for its competing overall performance and speed. The model was fine-tuned using a binary cross-entropy with logits loss, natively implemented in PyTorch [34]. The reason for opting for a multilabel text classification model was to mimic the behavior of a language model, whereby it outputs simultaneously both self-harm status and timing labels. For consistency, the RoBERTa model was trained and evaluated using the same training and test data splits used to develop the Gemma3 model. For reproducibility and detailed training, see Section B of Multimedia Appendix 1.

### Prompt Engineering

The effective use of language models for information extraction relies heavily on well-designed prompts that provide clear instructions and context for the task at hand. In this study, we focused on specifying rules and contextual cues to identify self-harm and determine its timing. Furthermore, our prompt design addressed the challenge of distinguishing actual self-harm events from related concepts such as suicidal ideation and self-harm risk. The prompt development followed established prompt engineering principles, eg, chain-of-thought and panel-of-experts approaches [35,36], using task decomposition [29] to address two sequential classification tasks: (1) binary detection of self-harm presence, and (2) temporal classification into 3 categories.

The prompt design incorporated clear inclusion criteria for completed intentional acts, comprehensive exclusion criteria (eg, thoughts, plans, and threats), and specific guidance for ambiguous cases. The final prompt required JSON-formatted output with direct textual evidence for each classification, using a 90-day threshold for determining a recent episode of self-harm. The prompt was validated by 2 self-harm experts before deployment.

To mitigate the risk of prompt overfitting and to meaningfully compare to a baseline RoBERTa model, the models were trained and tested on the same split partitions (Figure 2). For the Gemma3-27b iterative prompt refinement procedure, the training dataset was further split into development (n=542) and validation (n=542) datasets. The optimal prompt was developed iteratively on the development set (n=542), and after satisfactory performance was achieved on the validation set (n=542), the model was finally evaluated on the held-out test set (n=268).

For RoBERTa, we trained two variants: RoBERTa (n=542) using only the development set for fair comparison with Gemma3-27b’s prompt refinement data exposure, and RoBERTa (n=1084) using the full training set. Both models were evaluated on the identical held-out test set, ensuring unbiased performance comparison. The data flow through our experimental pipeline is illustrated in Figure 1. The prompt engineering approach with all development details and the prompt used in this work is presented in Section C of Multimedia Appendix 1.

### Ethical Considerations

This study received approval from the Oxford Health NHS Foundation Trust CRIS Oversight Committee and data were processed in accordance with the procedure outlined by the Oxford Health NHS Foundation Trust [37].

## Results

### Annotation Consistency

The results of inter-annotator agreement are shown in Table 2. These include CIs calculated using 1000 bootstrap resamples on a random set of 160 notes annotated independently by 2 human experts (GG and LB). The agreement was very good for self-harm status (presence) and was good for timing on self-harm-present notes [38], indicating robust and unambiguous annotation rules and the ability of 2 independent annotators (GG and LB) to follow it easily.

**Table 2.** Inter-annotator agreement for identifying self-harm and its timing.

Decision layer	Cohen κ (95% CI)
Self-harm status: present vs absent	*κ_self-harm_*=0.86 (0.78-0.94)
Timing of self-harm: recent vs historical vs unknown	*κ_recency_* = 0.71 (0.55-0.83)

### Multilabel Self-Harm Identification Models

Unless otherwise specified, we report *F*_1_-score. Sensitivity, specificity, and recall metrics are reported explicitly. Although Gemma32-7b requires no task-specific fine-tuning, we nevertheless built 2 supervised baselines to benchmark its zero-shot prompt- based extraction. The first, RoBERTa (n=542), was trained on the same 542- note development split that guided prompt refinement, giving a like-for-like comparison in terms of labeled data “ seen” by each approach. To assess the models’ performance, we computed the point estimates for precision, recall, and *F*_1_-score for each of the five categories: (1) at the self-harm status level: present and absent, and (2) at the timing level: recent self-harm episode, historical episode, and unknown timing. The performance metrics with corresponding 95% CIs are presented in Table 3.

**Table 3.** Comparisons of the three models’ performance according to self-harm status and its timing labels. Two baseline RoBERTa^a^ models were trained on datasets with 542 and 1084 samples.

Category (classification)	Model	Precision (95% CI)	Recall (95% CI)	*F*_1_-score (95% CI)
Self-harm (absence)	RoBERTa (n=542)	0.89 (0.86-0.92)	0.93 (0.90-0.95)	0.91 (0.89-0.93)
Self-harm (absence)	RoBERTa (n=1084)	0.92 (0.89-0.96)	0.98 (0.96-0.99)	0.95 (0.93-0.97)
Self-harm (absence)	Gemma3-27b (n=542)	0.96 (0.93-0.99)	0.97 (0.95-0.99)	0.97 (0.95-0.98)
Self-harm (presence)	RoBERTa (n=542)	0.81 (0.74-0.87)	0.72 (0.65-0.79)	0.76 (0.71-0.82)
Self-harm (presence)	RoBERTa (n=1084)	0.94 (0.88-0.98)	0.81 (0.71-0.89)	0.87 (0.80-0.92)
Self-harm (presence)	Gemma3-27b (n=542)	0.92 (0.86-0.98)	0.91 (0.86-0.95)	0.92 (0.88-0.96)
Timing (historical)	RoBERTa (n=542)	0.87 (0.10-1)	0.07 (0-0.18)	0.12 (0-0.31)
Timing (historical)	RoBERTa (n=1084)	0.65 (0.10-1)	0.11 (0-0.29)	0.18 (0-0.43)
Timing (historical)	Gemma3-27b (n=542)	0.47 (0.25-0.68)	0.53 (0.28-0.77)	0.51 (0.27-0.68)
Timing (recent)	RoBERTa (n=542)	0.52 (0.44-0.61)	0.75 (0.66-0.84)	0.62 (0.54-0.69)
Timing (recent)	RoBERTa (n=1084)	0.67 (0.56-0.79)	0.86 (0.76-0.94)	0.75 (0.65-0.84)
Timing (recent)	Gemma3-27b (n=542)	0.84 (0.79-0.89)	0.75 (0.59-0.90)	0.79 (0.65-0.89)
Timing (unknown)	RoBERTa (n=542)	0	0	0
Timing (unknown)	RoBERTa (n=1084)	0	0	0
Timing (unknown)	Gemma3-27b (n=542)	0.32 (0.07-0.62)	0.44 (0.11-0.68)	0.39 (0.09-0.61)
Average (micro)	RoBERTa (n=542)	0.80 (0.76-0.83)	0.78 (0.74-0.82)	0.79 (0.75-0.82)
Average (micro)	RoBERTa (n=1084)	0.85 (0.79-0.90)	0.84 (0.79-0.89)	0.83 (0.78-0.88)
Average (micro)	Gemma3-27b (n=542)	0.88 (0.84-0.91)	0.87 (0.84-0.91)	0.88 (0.84-0.91)
Average (weighted)	RoBERTa (n=542)	0.78 (0.72-0.82)	0.78 (0.72-0.82)	0.76 (0.72-0.80)
Average (weighted)	RoBERTa (n=1084)	0.85 (0.79-0.90)	0.84 (0.79-0.89)	0.83 (0.78-0.88)
Average (weighted)	Gemma3-27b (n=542)	0.88 (0.84-0.92)	0.87 (0.84-0.91)	0.88 (0.84-0.92)

a RoBERTa: robustly optimized BERT pretraining approach.

Table 3 shows that RoBERTa (n=542) underperformed Gemma3-27b across all labels, most notably on recent self-harm (*F*_1_=0.62 vs 0.79) and on the low-prevalence historical class, where recall collapsed to only 7% versus 53% that of Gemma3-27b. The multilabel confusion matrices for all categories are shown in Figure S1 in Section D of Multimedia Appendix 1.

We assumed that weak performance of RoBERTa could be attributed to limited training examples rather than intrinsic model capacity. Accordingly, we trained a second baseline RoBERTa (n=1084) on the full-1084 note training set. Performance improved, but even with nearly double the annotations RoBERTa still failed to surpass Gemma’s precision-recall balance, demonstrating that even relatively small (27 billion parameters) privacy-preserving language model offers a stronger starting point than a task-specific transformer, even when the latter is given all available data.

The Gemma3-27b model demonstrated superior performance across all classification categories, achieving a weighted *F*_1_-score of 0.88 and micro *F*_1_-score of 0.88, compared to RoBERTa’s 0.83 and 0.83, respectively. This is consistent with the broader pretrained knowledge that LLMs bring to semantically complex clinical tasks, although the aggregate gains were modest (≈3‐5 weighted *F*_1_ points). The disparity was particularly pronounced for the more challenging temporal categories. RoBERTa failed entirely to identify “unknown timing” cases (*F*_1_=0.0) and performed poorly on “historical” classifications (*F*_1_=0.18), while Gemma3-27b achieved *F*_1_-scores of 0.39 and 0.51 for these categories, respectively. This stark difference highlights a fundamental limitation of supervised approaches when training data are scarce. Both categories were rare in the corpus, with 77 of 1352 notes (5.7%) labeled as “unknown” and 90 of 1352 notes (6.7%) as “historical.” The performance of 2 leading models, Gemma3-27b and RoBERTa (n=1084), were compared using McNemar test for multilabel classifications with bootstrap analysis and the Benjamini-Hochberg false discovery rate method for multiple comparison correction. All details of the statistical comparison are shown in Section E of Multimedia Appendix 1.

The RoBERTa model’s difficulty with rare categories illustrates a well-known challenge in clinical NLP: obtaining sufficient annotated examples for every category is often impractical. Supervised learning typically requires many examples per class to achieve reliable performance, a requirement rarely met for infrequent but clinically important categories.

Gemma3-27b’s relatively stronger performance on these rare categories, achieved through iterative prompt engineering on a labeled development set rather than gradient-based training, suggests that the model’s pretrained knowledge provides a useful starting point for handling the long-tail distribution typical of clinical data. However, we note that this advantage was most evident in lower-frequency timing categories; aggregate gains over RoBERTa trained on the full dataset were modest. For reproducibility and technical details of model training, please refer to Section F of Multimedia Appendix 1.

### Binary Classification for Recent Self-Harm Detection

To evaluate real-world applicability, we reformulated our multilabel task as a binary classification problem focused on identifying recent self-harm, the most clinically actionable category.

**Figure 3.**
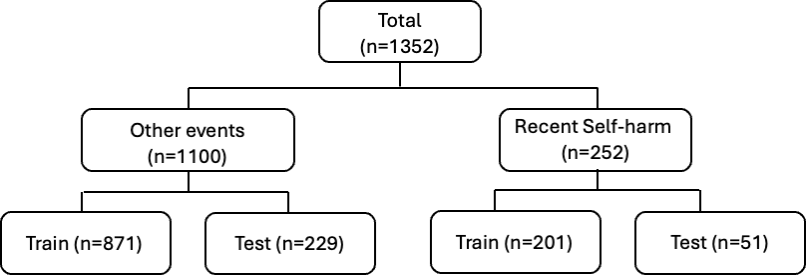
Binary relabeling of multilabel annotated examples.

This approach mirrors practical use cases where, for example, clinicians may need to identify patients requiring intervention. This approach provides a simplified and practical categorization, aimed at identifying individuals with a recent self-harm event. We combined the original labels into two categories: (1) “Recent self-harm”—cases with confirmed self-harm occurring within 90 days (n=252), and (2) “Other events”—all remaining cases, including absent self-harm, historical events, or unknown timing (n=1100), as shown in the data flow chart in Figure 3.

We maintained identical train-test splits to ensure fair comparison between a baseline RoBERTa and Gemma3-27b models. The baseline RoBERTa model was retrained from scratch using binary cross-entropy loss optimized for the new labels. For Gemma3-27b, we retained the original multilabel prompt, then programmatically converted its structured output: cases labeled as both “self-harm present” AND “recent” were classified as “Recent self-harm”; all other label combinations mapped to “Other events.” The results are presented in Table 4.

**Table 4.** Performance of two models identifying recent self-harm. The alternative category ‘Other events’ includes any combinations, such as unconfirmed self-harm, historical events of confirmed self-harm or self-harm where timing is unknown.

Category	Model	n cases	Precision (95% CI)	Recall (95% CI)	*F*_1_ (95% CI)
Recent self-harm	RoBERTa^a^	51	0.79 (0.68-0.90)	0.82 (0.71-0.92)	0.81 (0.71-0.88)
Recent self-harm	Gemma3-27b	51	0.77 (0.64-0.89)	0.72 (0.60-0.84)	0.74 (0.64-0.83)
Other events	RoBERTa	201	0.96 (0.93-0.98)	0.95 (0.92-0.98)	0.95 (0.93-0.97)
Other events	Gemma3-27b	201	0.94 (0.90-0.97)	0.95 (0.92-0.98)	0.94 (0.92-0.96)
Average (micro)	RoBERTa	—^b^	0.93 (0.89-0.96)	0.93 (0.89-0.96)	0.93 (0.89-0.96)
Average (micro)	Gemma3-27b	—	0.91 (0.87-0.94)	0.91 (0.87-0.94)	0.91 (0.87-0.94)
Average (weighted)	RoBERTa	—	0.93 (0.89-0.96)	0.93 (0.89-0.96)	0.93 (0.89-0.96)
Average (weighted)	Gemma3-27b	—	0.91 (0.87-0.94)	0.91 (0.87-0.94)	0.91 (0.87-0.94)

a RoBERTa: robustly optimized BERT pretraining approach.

b Not applicable.

The results confirmed that privacy-preserving language models can effectively identify time-sensitive clinical events. While both models demonstrated strong performance for identifying recent self-harm, the re-trained RoBERTa model achieved marginally better performance with an average weighted *F*_1_-score of 0.93 (95% CI 0.89-0.96) compared to Gemma3-27b ’s *F*_1_-score of 0.91 (95% CI 0.87-0.94). For the dominant “Other events” category, both models performed well (*F*_1_>0.94) with almost identical performance.

## Discussion

### Principal Findings

In this study, we aimed to evaluate whether privacy-preserving local language models could identify self-harm and its timing in secondary mental health records. The Gemma3-27b model, containing 27 billion parameters with a 128K context window, was quantized to 4-bit precision and deployed locally via Ollama, ensuring complete data privacy within the host health care provider’s secure data infrastructure (in our case, the National Health Service). In a corpus of 1352 mental health clinical notes, Gemma3-27b outperformed a fine-tuned RoBERTa classifier on both detection of self-harm events and assignment of self-harm timing labels. The absolute *F*_1_ gain was modest for event detection (≈4%‐6%) but substantial for challenging “historical” and “unknown” timing categories (gains of 33% and ≥39%, respectively). Performance on the “recent” category reached an *F*_1_-score of 0.79 without gradient-based fine-tuning, although this result depended on systematic prompt development using a labeled development set. The largest relative improvements over RoBERTa were observed in the rarer timing categories, while aggregate gains were modest.

On the binary task of identifying recent self-harm, RoBERTa achieved a marginally higher weighted *F*_1_-score (0.93) than Gemma3-27b (0.91), although both models performed strongly and were comparable on the dominant “Other events” class. This indicates that supervised classifiers can be highly effective when the classification task is simplified and sufficient labeled data exists. The relative advantage of the prompt-based approach is most evident in the multilabel temporal setting, particularly for rarer timing categories where supervised models struggle without large per-class annotation volumes.

### Comparison With Previous Work

Ayre et al [15] developed a hybrid rule-based NLP tool using spaCy to identify perinatal self-harm in EHRs from the South London and Maudsley NHS Foundation Trust, achieving micro-averaged *F*_1_-scores greater than 0.8 for span, polarity, and temporality detection. However, their approach required extensive manual feature engineering, custom tokenization rules, and lexicon development. Similar to our findings, they reported temporality as the most challenging attribute (*κ*=0.62) and successfully used a heuristic requiring 2 or more mentions for patient-level classification. While their rule-based system performed well, it required 13 manually curated lexicons and complex grammatical rules, highlighting the engineering burden of traditional NLP approaches. In contrast, our prompt-based Gemma3-27b achieved comparable or superior performance without task-specific feature engineering, demonstrating the efficiency gains of using modern LLMs. It is worth noting that both studies identified the same clinical challenge: the ambiguity in temporal expressions within clinical documentation, suggesting that this represents a fundamental limitation in how clinicians record self-harm events, rather than a purely technical challenge.

### Clinical and Public Health Implications

Accurate ascertainment of self-harm is crucial for improving self-harm surveillance, evaluating services, and testing new interventions designed to support people who self-harm. It is also vital for identifying individuals in need of support. As the Gemma3-27b model requires relatively low computing resources, it can be deployed on in-house GPUs within a health care provider’s secure data infrastructure. This mitigates concerns about the use of “as a service” proprietary language models hosted outside the provider’s own infrastructure where inference using prompting with patient-level data cannot be guaranteed to be consistent with relevant and territory-specific statutory regulations. The approach demonstrated here, namely locally developed and quantized language models deployed within a secure data environment, establishes the technical feasibility of privacy-preserving self-harm detection. Potential future applications include batch or near-real-time processing pipelines, clinical dashboards, and pseudonymized analytics. However, evaluation of operational feasibility, governance workflows, clinician-review safeguards, and scalability is beyond the scope of this study and would require dedicated implementation and prospective validation studies. While prompt-based approaches may reduce, though not eliminate, the need for large volumes of annotated training data and may facilitate adaptation to related clinical tasks such as method-specific self-harm detection, suicidal ideation, or protective factors, these extensions remain speculative and require empirical validation.

### Utility and Potential Applications of This Tool and Its Future Iterations

Self-harm is often imprecisely captured across settings, including in the United Kingdom. This tool can support efforts to improve the monitoring of self-harm within clinical populations where such information is recorded narratively. Reliable identification and tracking of self-harm over time can provide valuable insights into temporal trends of self-harm and help assess the impact of public health policies or broader societal events [39].

Such a tool could further facilitate the identification of individuals who have recently self-harmed and may be candidates for pharmacological or psychological interventions, enabling the recruitment of representative and diverse patient samples. Moreover, given that self-harm is a key outcome in mental health care, the tool can facilitate the extraction of such information to assess the impact of targeted interventions.

Systematic and reliable identification of self-harm in EHRs is also important for estimating the burden of self-harm within clinical settings, which is essential for service planning and the allocation of resources. Similarly, it can contribute to better quality self-harm research, especially where research questions require establishing the timing of self-harm to conduct longitudinal analyses.

### Qualitative Error Analysis

To improve transparency about model limitations, we conducted a qualitative examination of Gemma3-27b misclassifications on the held-out test set, identifying 5 recurring failure modes (detailed in Section G of Multimedia Appendix 1 with synthetic clinical-note examples constructed by the clinical team for governance compliance). These included false-positive self-harm detection, where templated risk-assessment language was mistaken for a confirmed act; false-negative detection, where self-harm described briefly within longer psychosocial narratives was overlooked; false-negative recency, where vague temporal expressions (eg, “a few weeks ago”) were defaulted to nonrecent despite falling within the 90-day window; false-positive recency, where present-tense clinical concern led the model to override explicit historical date markers; and false-positive unknown timing, where indirect but sufficient temporal cues (eg, age-based reasoning) were not integrated. A substantial proportion of these errors arose from genuine ambiguity in clinical documentation, contexts where even expert annotators required deliberation, rather than purely technical shortcomings. These findings highlight the importance of expert review of all model outputs prior to any operational deployment, and of continuous monitoring for potential data and model drift as documentation practices, clinical populations, or language model versions evolve.

### Strengths and Limitations

All computation occurred behind the Oxford Health NHS Foundation Trust firewall with no data egress, adhering to the relevant general data protection regulation and UK Data Security & Protection Toolkit standards. Two domain experts produced a high-quality gold standard with very good κ=0.86 for event detection. The study used identical splits and evaluation metrics for both the Gemma3-27b language model and the RoBERTa model, isolating the effect of model architecture. A single, relatively low-cost NC-T4 node (16 GB GPU) demonstrates broad feasibility across publicly funded health care settings, such as the NHS in the United Kingdom, without requiring large-scale high-performance computing infrastructure and a low carbon footprint.

This study has 6 main limitations. First, while demonstrating the feasibility of privacy-preserving language models for self-harm detection, we acknowledge limited model selection. We evaluated locally deployable models via Ollama (Llama 3.2, Mistral, Phi-4, Qwen 2, and various Gemma-3 variants), finding performance correlated with parameter count, consistent with established scaling laws [40,41]. Optimal prompts varied substantially across models, reflecting differences in pretraining, corpora, and architecture [42]. This model-specific sensitivity suggests Gemma3-27b may not represent optimal performance. We selected Gemma3-27b pragmatically as a representative high-performing model, balancing computational resources with demonstrating feasibility rather than identifying the optimal clinical deployment model. When using privacy-preserving, local quantized LLMs in applications similar to those in our study, it will be important to systematically evaluate different model architectures and prompting strategies.

Second, data for training and testing the models were sourced from a single region and secondary mental health care setting in England; therefore, external validity to other regions with different populations, or to primary and acute care settings, was not assessed. Of note, the nature of underlying data differs substantially between secondary mental health care (the data used in our study) and primary or acute care settings due to variations in clinical practice. For example, acute hospitals rely more heavily on structured clinical coding to record patient presentations and encounters involving self-harm, whereas mental health care data place greater emphasis on narrative psychosocial formulations of historical and current self-harm and its management. For these reasons, we would expect that, across the United Kingdom’s secondary mental health care system, the presented model would show limited variation in performance for the self-harm task described here, as services share a similar culture of practice and use EHRs with comparable functionality. However, a substantially different model would likely be required to address the same task in acute or primary care EHRs.

Third, a potential selection effect arises from using a Gemma3-4b model for candidate-note identification and a Gemma3-27b model as the primary evaluation model. Although these models differ substantially in parameter count (4 billion vs 27 billion) and the screening step was used solely to make annotation feasible, it did not generate gold-standard labels, and no model parameters were updated based on screening outputs, both models belong to the same architectural family. If Gemma-family models share systematic biases in what they flag as self-harm-related, the evaluation corpus could, in principle, contain a distributional signature that favors Gemma3-27b over architecturally different models such as RoBERTa. Two observations mitigate this concern: (1) the screening step removed many “easy negatives” (eg, administrative notes), yielding a harder evaluation set containing more ambiguous cases, which are consistent with the study’s clinical aims; and (2) RoBERTa achieved strong overall performance and marginally outperformed Gemma3-27b on the simplified binary task, which would not be expected under strong architectural bias. Nevertheless, a residual distributional effect cannot be excluded. Future validation should include sensitivity analyses, such as alternative screening strategies (eg, keyword-based or clinician-led) or a supplementary truly random annotated sample, to quantify the magnitude of any selection effect.

Fourth, because the annotated corpus is enriched for potential self-harm content, the class distribution does not reflect the prevalence that would be encountered when the model is deployed across all clinical notes in routine care. In a low-prevalence setting, even a model with high specificity can generate a nontrivial number of false positives at scale, increasing clinician review burden and potentially undermining trust. Prospective evaluation under true-prevalence conditions, prevalence-aware calibration or thresholding strategies, and clinician-in-the-loop workflows in which every model output is reviewed before any clinical action are essential prerequisites for operational deployment.

Fifth, this study did not evaluate operational deployment considerations. Questions relating to batch versus near-real-time processing architectures, governance frameworks for automated flagging, human-in-the-loop safeguards, and real-world performance under routine clinical conditions were beyond the scope of the present work and constitute essential future research.

Sixth, many patients had a long-documented history of contact with secondary mental health services. Although annotators were instructed to treat each clinical note as a standalone document, independent of any previous decisions made about previous extracts from the same patient, this may not have been fully achievable in practice. As a result, some annotations may have been influenced by broader impressions of the patient rather than by information explicitly present in the text being analyzed. Fourth, historical (90 of 1352, 6.7%) and unknown (77 of 1352, 5.7%) timing cases were underrepresented, inflating CIs despite bootstrap resampling. Future work should use active-learning strategies to enrich rare labels. Fifth, the 90-day threshold, while pragmatic, may not entirely align with all clinical use cases; finer-grained temporal consensus on recency remains challenging. However, the recency threshold could be readily changed by amending the prompt.

### Conclusions

This work demonstrates the technical feasibility of using a privacy-preserving, locally deployable language model within a secure NHS data infrastructure to identify self-harm and its timing. Without gradient-based fine-tuning, but with systematic prompt development on a labeled development set, Gemma3-27b matched or exceeded a fine-tuned RoBERTa classifier, with the largest gains in challenging, lower-frequency timing categories and modest aggregate improvements. On a simplified binary task, RoBERTa performed marginally better, highlighting that the choice of approach should be guided by the specific clinical task and available annotation resources. These findings establish a proof of concept; clinical deployment would require multicenter validation across geographically diverse areas and populations, prospective evaluation of operational workflows (including clinician-review safeguards, governance frameworks, and false-positive management), implementation research with stakeholders, and rigorous monitoring for model drift and unintended bias. Such studies are the critical next steps to translate privacy-preserving language models into improved self-harm surveillance and patient confidentiality.

## Supplementary material

10.2196/87586 Multimedia Appendix 1 Additional tables, figures, and information.

## Abbreviations

API: application programming interface
CRIS: Clinical Record Interactive Search
EHR: electronic health record
GPU: graphical processing unit
*ICD-10*: *International Statistical Classification of Diseases, Tenth Revision*
LLM: large language model
NHS: National Health Service
NLP: natural language processing
RoBERTa: robustly optimized BERT pretraining approach
