# Supplementary material for: Detection of Self-Harm in Electronic Mental Health Records Using Privacy-Preserving Local Language Models: Methodological Study
Source: JMIR Ment Health. 2026 Jun 2;13:e87586. doi: 10.2196/87586 (PMC13229841; doi:10.2196/87586)
Supplement: Multimedia Appendix 1 [file mental-v13-e87586-s001.docx]

A. Cohort statistics

**Table S1.** The annotated cohort description used to develop and validate models.

| **Characteristic** | **n (%) or median [IQR]** |
| --- | --- |
| ***Demographics*** | |
| Total clinical notes | 1,352 |
| Total unique patients | 628 |
| Ageᵇ, years | 37 [25.8, 53.3] |
| 18–24 | 151 (24) |
| 25–44 | 233 (37.1) |
| 45–64 | 191 (30.4) |
| ≥65 | 53 (8.4) |
| **Sex** |  |
| Female | 355 (56.5) |
| Male | 273 (43.4) |
| **Ethnicity** |  |
| White | 463 (84.2) |
| Asian | 23 (4.2) |
| Mixed | 20 (3.7) |
| Black | 11 (2.0) |
| Other | 5 (1.7) |
| Not stated/Unknown | 106 (16.9) |
| ***Top 10 Primary Mental Health Diagnoses (ICD-10)ᵃ*** | |
| **Psychotic disorders (F20–F29)** |  |
| F20 – Schizophrenia | 45 (9.9) |
| F25 – Schizoaffective disorders | 19 (3.8) |
| **Mood disorders (F30–F39)** |  |
| F31 – Bipolar affective disorder | 47 (9.5) |
| F32 – Depressive episode | 89 (17.9) |
| F33 – Recurrent depressive disorder | 34 (6.9) |
| **Neurotic/stress-related (F40–F48)** |  |
| F41 – Other anxiety disorders | 31 (6.3) |
| F43 – Reaction to severe stress | 22 (4.4) |
| **Behavioural syndromes (F50–F59)** |  |
| F50 – Eating disorders | 16 (3.2) |
| **Personality disorders (F60–F69)** |  |
| F60 – Specific personality disorders | 36 (7.3) |
| ***Self-harm History (from notes analysed)*** | |
| Any self-harm mentioned | 419 (30.9) |
| Recent (≤90 days) | 252 (18.6) |
| Historical (>90 days) | 90 (6.6) |
| Timing unclear | 77 (6) |
| ***Note Characteristics*** | |
| Notes per patient, median [IQR] | 2 [1, 3] |
| Note length (words), median [IQR] | 248 [146, 315] |

IQR = interquartile range.

Patients may have multiple diagnoses.

a Percentages based on unique patients (n=628).

b Age at first admission to secondary mental health care services.

B. Selection of clinical notes that may contain mentions of self-harm

We used a light-weight version of ‘Gemma-3’ model with 4 billion parameters to pre-select clinical notes that may contain mentions of self-harm without any further differentiation. The model was served via Ollama (0.9.6) framework using Python (3.12.8). The internal parameters of ‘Gemma3-4b’ were: temperature = 0, top_p = 0.95, top_k=64. Average end‑to‑end latency was 1.5 s per 500‑token note on a single Microsoft Azure ‘Standard_NC8as_T4_v3’ instances (NVIDIA T4 16GB).

Prompt text used to pre-select clinical notes:

###################### BEGINNING OF THE PROMPT ######################

### SYSTEM

You are an expert clinical‐text classifier.

Your only job is to decide whether the note you receive contains **any explicit or implicit mention of self‑harm** (suicidal ideation, self‑injury, suicide attempts, self‑poisoning, thoughts of wanting to die, etc.).

If the note contains even one such reference, answer **"YES"**.

If it does **not** (e.g., routine referrals, appointment letters, discharge summaries without self‑harm), answer **"NO"**.

### OUTPUT RULES

1. Output **only** a single JSON object that exactly matches this schema:

```json

{

"self_harm": "YES" | "NO",

"reasoning": "string"

}

### DEFINITIONS

– self_harm must be either "YES" or "NO" (uppercase).

– reasoning is a concise phrase up to 100 words justifying the label; do not expose chain‑of‑thought.

2. No additional keys, comments, markdown, or extra text, just the JSON.

### DECISION GUIDELINES

Label YES if the note includes any of the following (even once):

- Suicidal thoughts, plans or attempts

- Non‑suicidal self‑injury (cutting, burning, overdosing)

- Historical self‑harm that is clinically relevant to the note

- Risk assessments that state self‑harm risk/ideation is present

Label NO when:

- The note is administrative (appointment, referral, billing)

- It is a clinical summary with no self‑harm content

- Self‑harm is explicitly denied and no other self‑harm content appears

- Edge cases then err on the side of YES.

### EXAMPLES

INPUT NOTE A

“Patient reports cutting her forearm three days ago after argument with partner.”

OUTPUT

{"self_harm":"YES", "reasoning":"Recent self‑injury (cutting) described."}

INPUT NOTE B

“Follow‑up letter confirming physiotherapy appointment for lower‑back pain.”

OUTPUT

{"self_harm":"NO", "reasoning":"Administrative appointment; no self‑harm content."}

### TASK

Classify the following clinical note:

{note}

######################### END OF THE PROMPT #########################

C. Development of the self-harm detection prompt

We experimented with several approaches to capture nuanced information about self-harm episodes and their temporal characteristics. The development followed systematic design patterns recommended for distilled language models like Gemma3-27b. We developed comprehensive exclusion criteria through negative examples taken from clinical notes, including thoughts, plans, threats, and acts by others. Additionally, we included specific guidance for handling ambiguous scenarios and edge cases.

The initial prompt contained extensive instructions with multiple restatements of key concepts. While thorough, this approach suffered from several limitations: (i) lengthy (~1,200 words) with repeated instructions; (ii) scattered organisation lacking logical flow; (iii) inconsistent emphasis patterns, and (iv) absence of concrete examples.

We, therefore revised our approach and applied structured prompting principles to improve the prompt by introducing clear section headers for better navigation, grouped related instructions for coherence, paraphrasing repeated structures, added examples and precedence rules for disambiguation of multiple incidents (e.g., recent versus historical). In the final version, we expressed the temporal boundary as a precise 90-day threshold using mathematical notation. We introduced formal notation such as “RD” for record date to enhance clarity. Further, we adopted the following naming convention (e.g., “NOSH” stand for “NO Self-Harm”), following **Table S2.** Labelling convention used in this study.

| **Label** | **Naming convention** |
| --- | --- |
| Self-harm absent | SH_NOSH |
| Self-harm present | SH_SELFHARM |
| Recent | TM_RECENT |
| Historical | TM_HISTORICAL |
| Unknown timing | TM_UNKNOWN |

We strictly instructed the model to generate output in JSON format to ensure consistency, enhanced by using the Python’s `pydantic` class. The final prompt was reviewed by two experts in self-harm for its clinical correctness and consistency. To ensure reproducibility, the temperature parameter of the Gemma3-27b model was set to zero. All examples shown in the prompt below are made up by the clinical team according to data governance. Below is the final prompt used in this work.

###################### BEGINNING OF THE PROMPT ######################

### SYSTEM

You are a clinical-NLP assistant analysing patient records for self-harm.

### TASK

For the clinical note below (starts with [YYYY-MM-DD]), determine:

1. Did the patient perform an intentional act of self-injury or self-poisoning?

2. If yes, when did it occur?

### DEFINITIONS

Self-harm = Any COMPLETED intentional act of self-injury or self-poisoning by the patient, regardless of method or motive.

### CLASSIFICATION RULES

Label as SH_SELFHARM only if:

- Clear evidence of a completed act (e.g., "cut wrist", "took 20 tablets", "burned with cigarette")

- Conflicting information: If act clearly occurred, classify as SH_SELFHARM even if later denied

Label as SH_NOSH if:

- Thoughts, ideation, plans, or threats without action

- Risk assessments or statements about potential

- Acts by others (family, friends)

- Preparations without execution (e.g., "held pills but didn't take")

- No clear evidence of completed act

RECENCY (only if SH_SELFHARM)

Let RD = record date at start of note.

- TM_RECENT: Act occurred ≤90 days before RD OR uses phrases like "yesterday/last week/past month" OR describes ongoing behaviour

- TM_HISTORICAL: Act occurred >90 days before RD OR uses phrases like "last year/as a teenager"

- TM_UNKNOWN: Timing cannot be determined

- Priority: If multiple acts with different timing, return TM_RECENT

### OUTPUT

Return ONLY this JSON (no markdown, no explanations):

{

"self_harm": "SH_SELFHARM" or "SH_NOSH",

"recency": "TM_RECENT" or "TM_HISTORICAL" or "TM_UNKNOWN",

"method": "<brief description>" or "Unknown",

"evidence": "<exact quote>" or "Unknown",

"self_harm_date": "<date or time reference>" or "Unknown",

"record_date": "<YYYY-MM-DD from note>"

}

### EXAMPLE

Input: [2024-03-15] "Patient reports she cut her arms with a razor blade last Tuesday..."

Output:

{

"self_harm": "SH_SELFHARM",

"recency": "TM_RECENT",

"method": "cutting with razor blade",

"evidence": "cut her arms with a razor blade last Tuesday",

"self_harm_date": "last Tuesday",

"record_date": "2024-03-15"

}

Analyse this note:

{text}

######################### END OF THE PROMPT #########################

D. Model performance

Below are the multi-label confusion matrices of RoBERTa (n=1,084) (left column) and Gemma3-27b (right column) for each of the categories. Since presence and absence of self-harm are exclusive categories, both labels can be presented by a single matrix.

| **RoBERTa (n=1,084)** | **Gemma3-27b** |
| --- | --- |
| 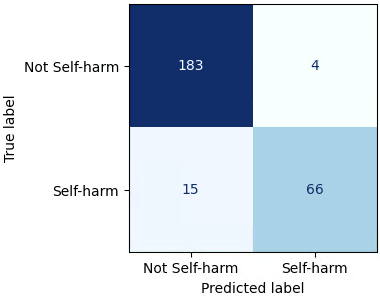 | 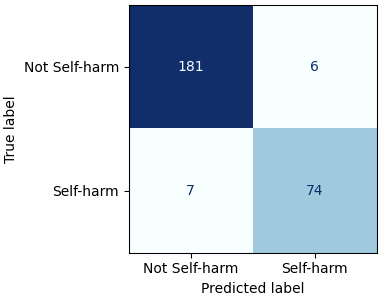 |
| 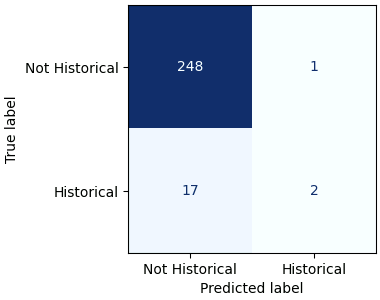 | 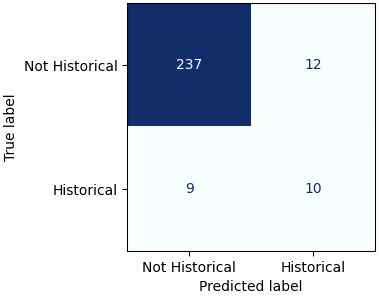 |
| 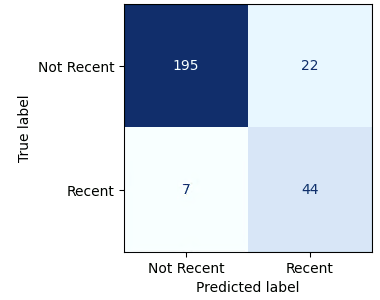 | 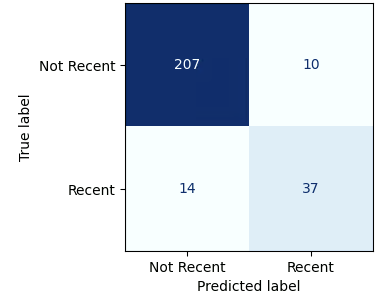 |
| 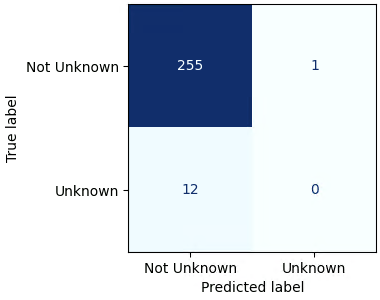 | 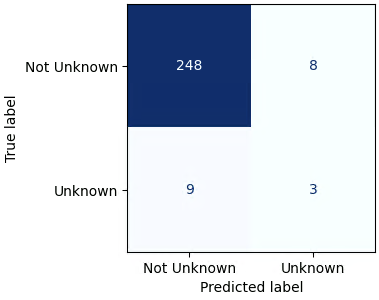 |

**Figure S1**. Comparison of multi-label confusion matrices for each of the categories.

Each matrix shows the performance of one specific label independently, showing how well the model predicts that particular (i.e., positive class) label. In these matrices, each label is treated as a binary classification problem, therefore, the “label” represents instances where the positive (predicted) class is present, while "not label" represents instances where that specific label is absent. Across categories, Gemma3-27b appears to be more balanced between false positive and false negative predictions, while RoBERTa captures more true positives as well as more false positives.

E. Statistical significance testing of model performance

Here we present detailed statistical comparisons between RoBERTa(n=1,084) and Gemma3-27b models using two-sided McNemar's test for multi-label classifications and bootstrap analysis of F1 scores. All tests were performed on the held-out test set n=268 with 10,000 bootstrap iterations with bias-corrected percentile intervals. Multiple comparison correction was applied using the Benjamini-Hochberg false discovery rate (FDR) method with α=0.05.

Binarisation of Multi-label Predictions

Since McNemar's test requires binary paired outcomes, we transformed our multi-label predictions using a one-vs-rest approach. For each of the five labels, we created binary predictions using the following algorithm (Table S3):

**Table S3:** Binarisation of multi-label schema for application of the McNemar’s test.

| Self-harm absent | 1 if predicted as absent, 0 otherwise |
| --- | --- |
| Self-harm present | 1 if predicted as present (regardless of timing), 0 otherwise |
| Recent | 1 if predicted as self-harm present AND recent, 0 otherwise |
| Historical | 1 if predicted as self-harm present AND historical, 0 otherwise |
| Unknown timing | 1 if predicted as self-harm present AND unknown timing, 0 otherwise |

Each McNemar's test compared these binarised predictions between models for a single label. The p-values in Table E.2 represent five independent tests, which is why we applied Benjamini-Hochberg correction for five comparisons (not multiple tests per label).

**Table S4:** Statistical comparison of RoBERTa(n=1,084) vs Gemma3-27b performance using McNemar's test and bootstrap analysis. ^a^ Number of cases where only RoBERTa vs only Gemma3-27b correctly predicted this label (binarised). ^b^ Difference = Gemma3-27b F1 - RoBERTa F1, calculated on binarised predictions for each label. ^c^ McNemar's test undefined when one model has zero unique correct predictions for this label. ^d^ Each row represents an independent McNemar's test on binarised predictions for that specific label. * Remains significant after FDR correction for 5 independent comparisons (one per label), bold indicates statistical significance at α=0.05.

The statistical analysis showed that Gemma3-27b significantly outperforms RoBERTa(n=1,084) on the most clinically challenging temporal classification tasks. McNemar's test showed significant differences for recent self-harm detection (χ²=10.00, p=0.007), with Gemma3-27b correctly identifying 18 cases missed by RoBERTa(n=1,084), while RoBERTa(n=1,084) uniquely identified only 10 cases. Bootstrap analysis with 10,000 iterations confirmed these findings and demonstrated the performance gains: (i) recent self-harm: Gemma3-27b achieved 16.8% higher F1 score (95% CI: 5.6-30.5%, p=0.006), (ii) historical self-harm: 32.5% improvement (95% CI: 5.5-47.8%, p=0.002) and (iii) unknown timing: 26.1% improvement (95% CI: 5.5-47.8%, p=0.039).

After Benjamini-Hochberg correction for multiple comparisons, the differences for recent (adjusted p=0.03) and historical (adjusted p=0.01) classifications remained statistically significant, while unknown timing became marginally non-significant (adjusted p=0.098).

RoBERTa(n=1,084) underperformed entirely to identify any historical or unknown timing cases that Gemma3-27b missed (0 unique correct predictions), demonstrating the supervised model inability to learn from rare categories despite training on the full dataset. This demonstrates the additional value of large language models pre-trained knowledge for handling clinical edge cases where training examples are scarce.

F. Reproducibility and details of models training

All experiments were conducted on Microsoft Azure ‘Standard_NC8as_T4_v3’ instances (NVIDIA T4 16GB). The baseline RoBERTa model, implemented in PyTorch (v2.4.1) using HuggingFace library (v4.54.0), was fine-tuned for multi-label classification using binary cross-entropy loss with logits. The base RoBERTa architecture contains 12 transformer layers, 768 hidden dimensions, and 12 attention heads (125M parameters). Training used AdamW (learning rate of 2 × 10⁻⁵, β₁ = 0.9, β₂ = 0.98), linear warm‑up over 500 steps with batch size = 8 for 10 epochs. One NVIDIA T4 16GB completed 10 epochs under 20 min.

The Gemma3-27b model, a decoder-only transformer containing 27 billion parameters with a 128K token context window, was quantized to 4-bit precision (Q4_K_M format) and converted to GPT-Generated Unified Format (GGUF), resulting in a 10.6 GB model size. The model employs a novel 5:1 local-to-global attention layer architecture with 1,024-token sliding windows for memory-efficient inference. It was served locally via the Ollama framework (v0.9.6) with llama.cpp backend. No gradient-based training was performed; instead, the model relied on zero-shot prompting with the carefully engineered prompt described in Appendix C.

For our experiments, we configured the context window to 8,192 tokens to balance performance and computational resources. Deterministic decoding (temperature=0, top_p=0.95 and top_k=64) produced the required JSON output which was parsed to extract labels. Average end-to-end latency was 90 seconds per 500-token clinical note on a single NVIDIA T4 16GB GPU served via Microsoft Azure.

All codes, annotated data and trained models can be made available upon request for users that are authorised on the Akrivia Health research platform.

G. Qualitative analysis of failure modes

To improve transparency about model limitations, we examined the major recurring patterns of misclassification ("failure modes") produced by Gemma3-27b on the held-out test set. For each failure mode we describe the common pattern and provide a synthetic clinical note excerpt that illustrates the type of language involved. All examples below were constructed by the clinical team and do not reproduce patient text.

## 1. False-positive self-harm - the model predicted a self-harm event where the gold standard confirmed that no actual act took place.

**Common pattern:** The model interpreted risk-assessment language, documentation of self-harm ideation, or references to historical self-harm within templated safety-plan fields as evidence of a completed act. Clinical notes that discuss self-harm potential or past self-harm purely in the context of ongoing risk monitoring, without describing a new or specific act, were particularly prone to this error.

**Synthetic example:** [2023-06-14] Review in community team. Risk assessment updated. History of self-harm by overdose, last known episode approximately two years ago. Current risk: moderate. Patient denies any recent self-harm or suicidal ideation. Plan: continue fortnightly contact, crisis plan reviewed.

Here, the note documents historical self-harm within a risk assessment, but explicitly states there has been no recent act. The model incorrectly classified this as self-harm present, apparently triggered by the phrase "self-harm by overdose" despite the negating context.

## 2. False-negative self-harm - the model missed a confirmed self-harm event by predicting its absence.

***Common pattern:****The model failed to recognise self-harm when the clinical language was indirect, euphemistic, or embedded within a longer narrative about the patient's psychosocial circumstances. Notes where the self-harm act was described briefly alongside extensive discussion of stressors, mental state, or treatment plans were particularly affected, as though the surrounding context diluted the salience of the event.*

***Synthetic example:*** [2024-01-22] Home visit. Patient appeared low in mood, described ongoing difficulties with housing and finances. Mentioned she had taken a handful of her prescribed tablets last Thursday following an argument, felt unwell but did not seek medical attention. Discussed safety planning and coping strategies. Will refer to psychology.

The note contains a clear self-harm act ("taken a handful of her prescribed tablets"), but the model classified it as self-harm absent, apparently because the overdose was mentioned briefly within a longer passage focused on social stressors and care planning.

3. False-negative recency - a recent self-harm event was misclassified as historical or of unknown timing.

**Common pattern:** When clinical text used vague or relative temporal language, such as "a few weeks ago," "recently," or "not long ago", without an explicit date, the model tended to default to a non-recent classification. The absence of a precise date appeared to be treated as evidence of temporal distance rather than as insufficient information. This was most pronounced in notes where the temporal expression was syntactically separated from the description of the act.

***Synthetic example:*** [2024-05-10] Outpatient review. Patient disclosed that she had cut her arms a few weeks ago during a period of distress. Wounds have healed. Currently feels more stable. No active suicidal ideation.

The self-harm occurred "a few weeks ago" relative to a note dated 10 May 2024, placing it well within the 90-day recency window. The model classified this as historical, apparently interpreting the past tense and the healed wounds as markers of a remote event.

4. False-positive recency - a historical or unknown-timing event was misclassified as recent self-harm.

**Common pattern:** The model incorrectly assigned recent timing when a note described current suicidal ideation, current emotional distress, or active clinical risk alongside a self-harm event that was explicitly dated beyond the 90-day window. The model appeared to apply a proximity heuristic: when any self-harm-related content co-occurred with present-tense clinical concern, it defaulted to "recent" regardless of the temporal markers attached to the act itself.

***Synthetic example:* [**2024-09-03] CPA review. Patient reports ongoing low mood and fleeting thoughts of self-harm. Previously took an overdose of paracetamol in January 2024, requiring A&E attendance. Currently engaging with crisis team. Risk assessment updated.

The overdose is explicitly dated to January 2024, more than seven months before the note, and should be classified as historical. The model classified it as recent, apparently influenced by the present-tense language about ongoing ideation and crisis-team involvement.

5. False-positive unknown timing - a self-harm event with determinable timing (recent or historical) was misclassified as unknown timing.

**Common pattern:** The model defaulted to "unknown timing" when the temporal information, although present, was conveyed indirectly or required inference from multiple cues rather than a single explicit date. Notes containing phrases such as "last year" or "when she was a teenager", which provide sufficient information to classify an event as historical, were sometimes labelled as unknown, suggesting the model applied an overly conservative threshold for temporal certainty rather than integrating contextual evidence.

***Synthetic example:*** [2024-07-18] Assessment. Patient reports that she used to cut herself regularly as a teenager; stopped around age 19. She is now 34. Scars visible on both forearms. No self-harm for many years.

The note establishes that self-harm occurred approximately 15 years ago, clearly historical. The model classified the timing as unknown, apparently because no specific date was given, despite the age-based temporal reasoning providing sufficient information.

These failure patterns highlight that a substantial proportion of misclassifications arise from genuine ambiguity or indirectness in clinical documentation, contexts where even expert annotators required deliberation. The findings highlight the importance of expert review of all model outputs prior to any operational deployment, and of continuous monitoring for potential data and model drift as documentation practices, clinical populations, or language model versions evolve.
